# Apolipoprotein E Polymorphism and Reproductive Patterns Among Postreproductive Women

**DOI:** 10.1002/ajhb.70260

**Published:** 2026-04-18

**Authors:** Paula Bartecka, Andrzej Galbarczyk, Magdalena Klimek, Ilona Nenko, Grazyna Jasienska

**Affiliations:** ^1^ Department of Environmental Health, Institute of Public Health, Faculty of Health Sciences Jagiellonian University Medical College Krakow Poland; ^2^ Doctoral School of Medical and Health Sciences Jagiellonian University Medical College Krakow Poland; ^3^ Lise Meitner Research Group BirthRites—Cultures of Reproduction Max Planck Institute for Evolutionary Anthropology Leipzig Germany

**Keywords:** antagonistic pleiotropy, Apolipoprotein E, fertility, genetic polymorphism, reproduction

## Abstract

**Objectives:**

Traits that are detrimental for health may persist in populations because they are advantageous for reproduction. Apolipoprotein E is a protein involved in lipid metabolism, and it is encoded by a polymorphic gene (*ApoE*) with three alleles: *ApoE2*, *ApoE3*, and *ApoE4*. *ApoE4* allele is associated with elevated cholesterol levels, and increased risk of various metabolic and age‐related diseases, such as cardiovascular disease and dementia. Because lipids are crucial for steroid hormone synthesis and thus the ovarian function, *ApoE4* allele may be associated with enhanced fertility. Therefore, we hypothesize that women with different *ApoE* genotypes will exhibit differences in reproductive history traits.

**Methods:**

Participants included 360 postreproductive women aged 45–92 from a Polish rural population living at the Mogielica Human Ecology Study Site. General linear models were used to test differences in age at menarche, age at first reproduction, number of children born, mean interbirth interval and age at last reproduction across different *ApoE* genotypes.

**Results:**

No significant differences were observed between *ApoE* genotypes in any of the tested reproductive history parameters.

**Conclusion:**

Although some of the previous research has suggested that carriers of *ApoE4* have more successful reproduction, we found no evidence supporting such an association among postreproductive aged women from a traditional, agricultural community. It is possible that *ApoE4* may confer reproductive advantages only under specific ecological or lifestyle conditions, such as high pathogen burden or low‐energy diet.

## 1 Introduction

Antagonistic pleiotropy is a phenomenon where a single gene has both positive and negative effects, often benefiting an organism early in life, while being detrimental in older age (Carter and Nguyen 2011; Hamilton 1966; Medawar 1946; Williams 2001). The *ApoE* gene encodes apolipoprotein E (APOE), a protein involved in lipid metabolism and neural function (Farrer et al. 1997; Hamilton 1966; Medawar 1946; Williams 2001). *ApoE* is located on chromosome 19 and has three alleles: *ApoE2, ApoE3, ApoE4* (Davignon et al. 1988; Niu et al. 2009). The structural differences among the *ApoE* alleles lead to differences in the structure of amino acids and the function of the protein (Farrer et al. 1997).

*ApoE4* allele is related to high cholesterol level due to more efficient absorption from the intestine, *ApoE2* is linked to reduced absorption, while *ApoE3* is considered neutral for cholesterol level (Bennet et al. 2007). Carriers of the *ApoE4* allele face an elevated risk of various metabolic and age‐related diseases (Mahley et al. 1996), including cardiovascular conditions such as coronary heart disease (Bennet et al. 2007; Shao et al. 2022), atherosclerosis (Lin et al. 2024), ischemic heart disease (Shi et al. 2018; Song et al. 2004), hypertension (Shi et al. 2018), as well as dementia (Rasmussen et al. 2020), most commonly resulting from neurodegenerative disorders such as Alzheimer's disease (Aita et al. 2024; Hersi et al. 2017). *ApoE4* is considered the ancestral form of the gene, with *ApoE3* emerging approximately 220 000 years ago, followed by *ApoE2* (Fullerton et al. 2000). Although there is substantial evidence supporting the detrimental health effects of *ApoE4*, this allele remains relatively common in many populations.

Allele frequencies differ among populations. In Polish population, *ApoE3* is the most common allele with frequencies ranging from 81.8% to 86.0%, the *ApoE4* allele occurs much less frequently (9.3%–10.6%), while *ApoE2* is the rarest (4.7%–7.6%) (Bednarska‐Makaruk et al. 2001; Jasienska et al. 2015; Kowalska et al. 1998). These ranges are broadly consistent with patterns observed in other European populations, for example, in Italian population allele frequencies of *ApoE3* are 83.1%–87.1%, *ApoE4* 7.5%–7.6%, and *ApoE2* 5.3%–9.2% (Corbo, Scacchi, et al. 2004), and in Danish population frequency of *ApoE3* is 74.6%, *ApoE4* 16.6%, and *ApoE2* 8.8% (Gerdes et al. 1996). Native American studies show that while *ApoE3* is much more common (90%–91.5%) than *ApoE4* (8.4%–10%), *ApoE2* is absent (Gamboa et al. 2000). Interestingly, the frequency of *ApoE4* is relatively high in several populations, including African‐Ecuadorians (23%–39%) and Cayapa Indians (32%) (Corbo, Ulizzi, et al. 2004), Ghanian population (12.5%–15.7%) (van Exel et al. 2017) and Tsimane tribe in Bolivian Amazon forest (~11%) (Trumble et al. 2023), reflecting diverse evolutionary histories and potential adaptive benefits of this allele in different environments.

Despite its well‐documented negative effects on health in later life, the *ApoE4* allele is thought to be maintained in human populations due to potential reproductive benefits that its carriers have (Bartecka and Galbarczyk 2026; Corbo et al. 2008). Our previous study showed that in a Polish population, young women (*N* = 117) carrying at least one copy of the *ApoE4* allele had higher levels of progesterone (Jasienska et al. 2015), a hormone essential for successful embryo implantation in the uterus and for pregnancy maintenance (Norwitz et al. 2001). This suggests that women carrying the *ApoE4* allele could experience higher fertility (Jasienska et al. 2015).

Several studies have examined the relationship between *ApoE* polymorphism and the number of children born to a woman. For instance, a study conducted in a Ghanaian population (*N* = 4311) confirmed that *ApoE4* was associated with increased fertility, but only among individuals exposed to high levels of pathogens (van Exel et al. 2017). In contrast, research conducted in European populations (Italian, *N* = 160; Danish, *N* = 379) has shown that carriers of the *ApoE3* allele had a higher number of children (Gerdes et al. 1996), while in an Ecuadorian population (*N* = 84), women with the *ApoE3/ApoE4* genotype were found to have higher fertility than those with other genotypes (Corbo, Ulizzi, et al. 2004). Moreover, an animal model study (*N* = 6) indicated that the number of offspring and litter sizes did not differ between *ApoE*‐knockout mice and controls (Zhang et al. 2014). Most human studies have focused primarily on the number of children as a measure of fertility, with only one study (*N* = 795) exploring other aspects of reproductive history and reporting that *ApoE4* carriers have an earlier age at first reproduction and shorter interbirth intervals (Trumble et al. 2023).

The aim of this study was to examine whether apolipoprotein E gene polymorphism was associated with fertility of women who have completed their reproduction. Based on previous evidence that *ApoE4* carriers exhibit higher progesterone levels during the menstrual cycle (Jasienska et al. 2015), we hypothesized that this genotype may be linked to higher reproductive success. Unlike most previous studies, which have focused primarily on the number of children, this research considered a broader set of reproductive history characteristics. Specifically, we tested the hypothesis that *ApoE4* carriers will have a higher number of children, an earlier age at menarche and at first reproduction, a shorter mean interbirth interval, and a later age at last reproduction. In addition, we explore the differences in reproductive parameters between women with various *ApoE3* allele combinations, as some studies (Corbo, Scacchi, et al. 2004; Gerdes et al. 1996) have suggested that genotypes with this allele may be associated with a higher fertility, particularly in European populations.

## 2 Materials and Methods

### 2.1 General Information

The research was conducted between 2011 and 2014 within an agricultural community living at the Mogielica Human Ecology Study Site, which includes several neighboring villages in southern Poland (Jasienska et al. 2015; Jasienska and Ellison 2004). This population was historically characterized by high parity and had limited access to modern contraception for an extended time (Colleran and Mace 2015). Additionally, self‐sufficient food production (by farming and livestock breeding) ensured relatively high energy intake (Jasienska 2013; Jasienska et al. 2006; Nenko and Jasienska 2009; Ziomkiewicz et al. 2008). Trained study assistants conducted household visits, inviting postreproductive women (aged 45 and above) to participate in the study. Additionally, the study was advertised by local churches and health clinics. No other exclusion criteria (apart from age below 45 years) were adopted at the time of data collection. The study was performed in accordance with the Declaration of Helsinki. All participants provided written, informed consent. The study received approval from the Jagiellonian University Bioethics Committee (KBET/5/B/2010 and KBET/328/B/2012).

### 2.2 Collected Data

**FIGURE 1:**
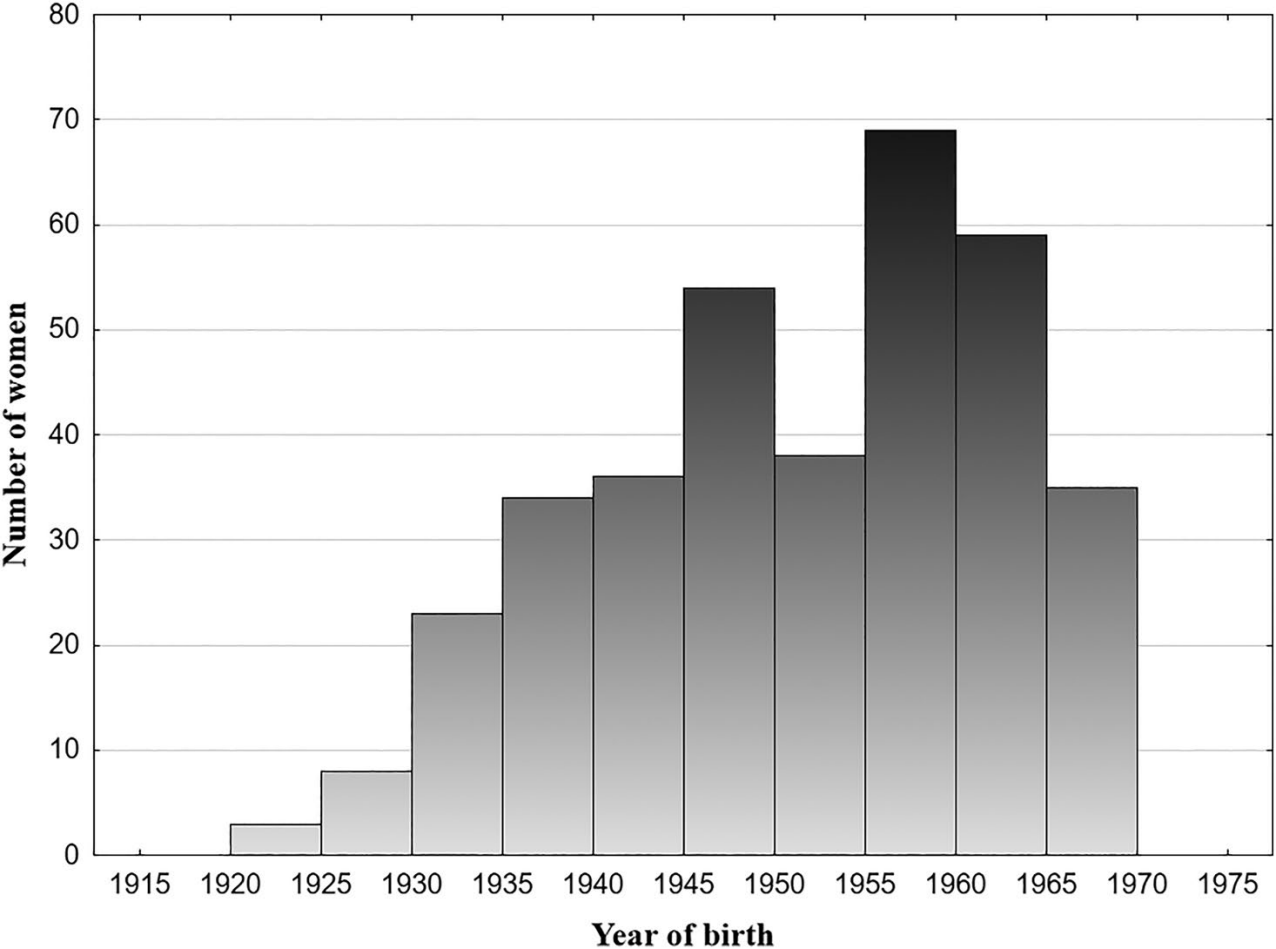
Distribution of year of birth among women in the study sample.

Reproductive history information was collected through a personalized questionnaire and birth records. The gathered data included the age at menarche, dates of all marriages, number of biological and adopted children, children's birth dates, date of death of a spouse or year of divorce (if occurred), and age at menopause (if occurred). For participants who provided complete birth date information for their children, calculations were made for age at first and last reproduction, as well as the mean interbirth interval (average number of months between consecutive births). Additionally, year of birth of a woman (Figure 1), and number of years of her education (as a proxy of socioeconomic status) (Colleran et al. 2014, 2015) were also collected and incorporated in the statistical analyses. Body height was measured using a stadiometer in the standardized position, without shoes, with an accuracy of 1 mm. Body weight was measured by digital scale (Tanita BC‐545N, Tokyo, Japan), with participants standing barefoot on the scale.

The initial study group included 432 women. To ensure that the analysis focused on individuals with uninterrupted reproductive potential, 62 participants were excluded based on specific criteria. We excluded women who were unmarried, divorced, widowed, or had experienced menopause before reaching the age of 45, or had at least one adopted child. Additionally, 10 women were excluded due to incomplete data such as age at marriage or age at divorce. Consequently, the final study sample consisted of 360 women, born between 1921 and 1969 (Figure 1), aged 45–92 (mean = 61.9, SD = 11.05) at the time of data collection. On average, these women had 4.1 children (SD = 2.11, range = 0–13). Detailed descriptive statistics for the study group are presented in Table 1. The number of participants included in different statistical models may vary due to missing data (Table 1).

**TABLE 1:** Descriptive statistics of the studied group.

Variable	*N*	Mean	Range		SD
Year of birth	360	1951.5	1921	1969	11.05
BMI (kg/m^2^)	359	30.1	18.8	46.7	5.26
Education (years)	357	10.1	3.0	23.0	3.36
Age at marriage (years)	359	23.3	16.8	42.6	4.17
Age at menarche (years)	336	14.6	10.0	19.0	1.52
Number of children	360	4.1	0.0	13.0	2.11
Age at first reproduction (years)	348	23.8	16.9	41.6	3.75
Age at last reproduction (years)	346	33.7	17.5	48.4	5.39
Mean interbirth interval (months)	331	41.5	11.6	160.5	20.15

### 2.3 Genotyping

**FIGURE 2:**
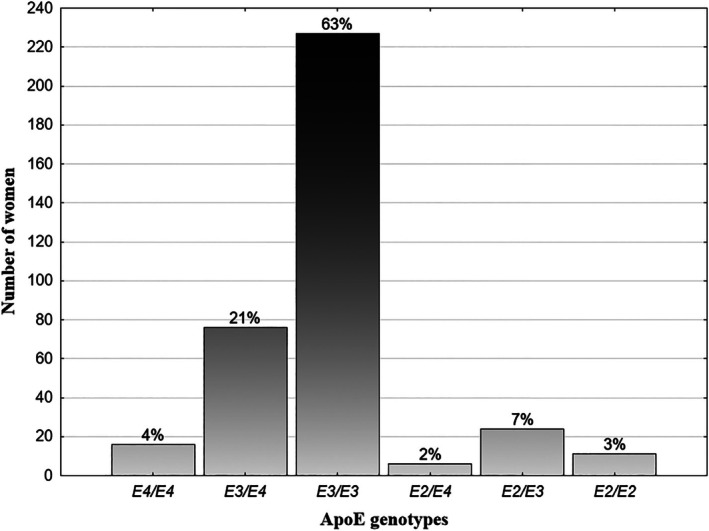
*ApoE* genotype frequencies in the study group.

Blood samples were collected at the local health clinics by professional personnel. Venous blood was drawn from the antecubital vein and immediately frozen at −80°C. Genotyping of the apolipoprotein E (*ApoE*) gene was performed using the polymerase chain reaction with sequence‐specific primers (PCR‐SSP) method, as detailed by Jasienska et al. (2015). Among the participants, the most common genotype was *ApoE3/ApoE3* observed in 227 women (63%), while the *ApoE2/ApoE4* genotype was the least common and was found in six women (2%; Figure 2).

### 2.4 Statistical Analyses

The data was analyzed using the Statistica version 14.1.0.4, employing the general linear model (GLM) to examine the association between *ApoE* genotype and reproductive outcome, while controlling for relevant covariates based on literature. Models with age at menarche were controlled for year of birth, number of years of education, models with age at first reproduction were controlled for year of birth, number of years of education, and age at marriage, and models with number of children, mean interbirth interval, and age at last reproduction were controlled for year of birth, number of years of education, and age at first birth. In the first step, women were divided in three groups based on presence of *ApoE4* allele: those with no *ApoE4* allele (No *ApoE4*), one *ApoE4* allele (One *ApoE4*), and two alleles of *ApoE4* (Two *ApoE4*). A similar categorization was applied for the *ApoE3* alleles. Subsequently, women were divided into two groups—those with at least one *ApoE4* allele (Any *ApoE4*), and those without this allele (No *ApoE4*), and then into those with at least one *ApoE3* allele (Any *ApoE3*), and those without this allele (No *ApoE3*).

## 3 Results

Women carrying *ApoE4* alleles did not differ in age at menarche, timing of reproduction, number of children, or mean interbirth interval. No statistically significant differences were observed between women with none, one, or two ApoE4 alleles (all *p* > 0.234; $\eta_{p}^{2}$ < 0.009) (Table 2). Similarly, when women were grouped by the presence of at least one *ApoE4* allele, there were no significant differences in their reproductive outcomes (all *p* > 0.115; $\eta_{p}^{2}$ < 0.008) (Table 3).

**TABLE 2:** Comparison of parameters of reproductive history among groups of women with none, one, or two *ApoE4* alleles. Differences among genotypes were tested by general linear model.

Reproductive characteristic	No *ApoE4*, *n* = 262, adjusted mean (SE)	One *ApoE4*, *n* = 82, adjusted mean (SE)	Two *ApoE4*, *n* = 16, adjusted mean (SE)	*F*	*p*	$\eta_{p}^{2}$
Age at menarche (years)^a^, ^b^	14.5 (0.11)	14.6 (0.18)	14.7 (0.42)	0.047	0.954	< 0.001
Age at first reproduction (years)^a^, ^b^, ^c^	23.8 (0.23)	23.8 (0.47)	23.8 (1.07)	0.275	0.760	0.002
Number of children^a^, ^b^, ^d^	4.2 (0.12)	4.3 (0.25)	4.8 (0.78)	0.037	0.964	< 0.001
Mean interbirth interval (months)^a^, ^b^, ^d^	42.6 (1.41)	39.3 (1.72)	34.8 (3.17)	1.457	0.234	0.009
Age at last reproduction (years)^a^, ^b^, ^d^	33.8 (0.34)	33.6 (0.60)	33.7 (1.80)	0.568	0.567	0.003

*Note:* Adjusted mean (SE)—mean value with standard error, $\eta_{p}^{2}$—partial eta squared.

^a^ Covariates: year of birth.

^b^ Covariates: education.

^c^ Covariates: age at marriage.

^d^ Covariates: age at first birth.

**TABLE 3:** Comparison of parameters of reproductive history between groups of women with none and at least one *ApoE4* allele. Differences between genotypes were tested by general linear model.

Reproductive characteristic	No *ApoE4*, *n* = 262, adjusted mean (SE)	Any *ApoE4*, *n* = 98, adjusted mean (SE)	F	*p*	$\eta_{p}^{2}$
Age at menarche (years)^a^, ^b^	14.5 (0.10)	14.6 (0.17)	0.052	0.820	< 0.001
Age at first reproduction (years)^a^, ^b^, ^c^	23.8 (0.23)	23.8 (0.43)	0.007	0.932	< 0.001
Number of children^a^, ^b^, ^d^	4.2 (0.12)	4.4 (0.25)	0.068	0.794	< 0.001
Mean interbirth interval (months)^a^, ^b^, ^d^	42.6 (1.42)	38.6 (1.53)	2.495	0.115	0.008
Age at last reproduction (years)^a^, ^b^, ^d^	33.8 (0.34)	33.6 (0.58)	0.445	0.505	0.001

*Note:* Adjusted mean (SE)—mean value with standard error, $\eta_{p}^{2}$—partial eta squared.

^a^ Covariates: year of birth.

^b^ Covariates: education.

^c^ Covariates: age at marriage.

^d^ Covariates: age at first birth.

Moreover, the *ApoE3* genotype was not associated with reproductive history traits. Comparisons among genotypes with none, one, or two *ApoE3* alleles revealed no significant differences in any of the reproductive traits assessed (all *p* > 0.221; $\eta_{p}^{2}$ ≤ 0.009) (Table 4). Furthermore, comparisons between *ApoE3* carriers and noncarriers revealed the absence of significant differences in reproductive characteristics (all *p* > 0.317; $\eta_{p}^{2}$ ≤ 0.003) (Table 5). A secular trend was observed regarding the number of children and the age at last birth within the study group; both the number of children and the maternal age at last reproduction decreased over time (see Supporting Information Table S1–S4 for GLM estimates regarding all effects).

**TABLE 4:** Comparison of parameters of reproductive history among groups of women with none, one, or two *ApoE3* alleles. Differences among genotypes were tested by general linear model.

Reproductive characteristic	No *ApoE3*, *n* = 33, adjusted mean (SE)	One *ApoE3*, *n* = 100, adjusted mean (SE)	Two *ApoE3*, *n* = 227, adjusted mean (SE)	F	*p*	$\eta_{p}^{2}$
Age at menarche (years)^a^, ^b^	14.8 (0.30)	14.5 (0.16)	14.6 (0.10)	0.365	0.694	0.002
Age at first reproduction (years)^a^, ^b^, ^c^	23.5 (0.78)	23.6 (0.40)	24.0 (0.24)	0.001	0.999	< 0.001
Number of children^a^, ^b^, ^d^	4.4 (0.45)	4.2 (0.21)	4.3 (0.13)	0.311	0.733	0.002
Mean interbirth interval (months)^a^, ^b^, ^d^	39.6 (3.72)	39.0 (1.71)	42.9 (1.50)	1.514	0.221	0.009
Age at last reproduction (years)^a^, ^b^, ^d^	33.4 (1.13)	33.1 (0.56)	34.0 (0.36)	1.294	0.276	0.008

*Note:* Adjusted mean (SE)—mean value with standard error, $\eta_{p}^{2}$—partial eta squared.

^a^ Covariates: year of birth.

^b^ Covariates: education.

^c^ Covariates: age at marriage.

^d^ Covariates: age at first birth.

**TABLE 5:** Comparison of parameters of reproductive history between groups of women with none and at least one *ApoE3* allele. Differences between genotypes were tested by general linear model.

Reproductive characteristic	No *ApoE3*, *n* = 33, adjusted mean (SE)	Any *ApoE3*, *n* = 327, adjusted mean (SE)	*F*	*p*	$\eta_{p}^{2}$
Age at menarche (years)^a^, ^b^	14.8 (0.30)	14.5 (0.09)	0.487	0.486	0.001
Age at first reproduction (years)^a^, ^b^, ^c^	23.5 (0.78)	23.8 (0.21)	0.001	0.974	< 0.001
Number of children^a^, ^b^, ^d^	4.4 (0.45)	4.2 (0.11)	0.457	0.500	0.001
Mean interbirth interval (months)^a^, ^b^, ^d^	39.6 (3.72)	41.7 (1.17)	0.295	0.587	0.001
Age at last reproduction (years)^a^, ^b^, ^d^	33.4 (1.13)	33.8 (0.30)	1.006	0.317	0.003

*Note:* Adjusted mean (SE)—mean value with standard error, $\eta_{p}^{2}$—partial eta squared.

^a^ Covariates: year of birth.

^b^ Covariates: education.

^c^ Covariates: age at marriage.

^d^ Covariates: age at first birth.

## 4 Discussion

We investigated the association between apolipoprotein E gene polymorphism and reproductive history of postreproductive women from a rural Polish population with high variation in fertility. We examined five reproductive history parameters, including age at menarche, timing of reproduction (age at first and last birth), number of children, and mean interbirth interval, which provided a comprehensive view of lifelong fertility. We have shown no differences in fertility parameters between women carriers of *ApoE4* and *ApoE3*, and non‐carriers. This suggests that potentially other factors, rather than *ApoE* gene, may play a more substantial role in influencing fertility patterns in this population.

We observed no differences in the number of children or mean interbirth interval among women with different *ApoE4* and *ApoE3* allele combinations. These findings align with a study in rural Ghana, where *ApoE4* was not associated with the number of children among women with low pathogen exposure (van Exel et al. 2017). Such findings support the idea that the reproductive advantages of *ApoE4* may be context‐dependent, emerging only under specific environmental or ecological pressures. Most of the studies have shown that there is an association between having *ApoE4* allele and higher fertility. For example, in Ghana, among women with higher pathogen exposure (i.e., who were taking water from open wells and rivers instead of borehole well water), those who were carrying one *ApoE4* alleles had nearly 3.5 times more children compared to those without this allele (van Exel et al. 2017). In a rural South American community Tsimane in Bolivian Amazon forest, women carrying at least one *ApoE4* allele had from 0.3 to 0.5 more children compared to those with the *ApoE3/ApoE3* genotype, while women with two *ApoE4* alleles showed an even greater increase in fertility, ranging from 1.4 to 2.1 more children (Trumble et al. 2023). In the same population, women carrying *ApoE4* allele achieved pregnancy 1.5 times more frequently, having shorter interbirth intervals by 0.23 years (Trumble et al. 2023). A study in a preindustrial population in Ecuador (Afro‐Ecuadorian and Cayapa Indians) revealed that heterozygotic women with the *ApoE3/ApoE4* genotype had, on the average, 1.5 times more children than those with the other genotypes (Corbo, Ulizzi, et al. 2004). By contrast, a study conducted among rural Italian population showed that only carrying the *ApoE3* was associated with a higher number of children. Women with the *ApoE3/ApoE3* genotype had, on the average, of 1.5 times more children compared to the *ApoE2/ApoE2*, *ApoE3/ApoE2*, and *ApoE4/ApoE3* genotypes (Corbo, Scacchi, et al. 2004; Corbo et al. 2008).

In our study, *ApoE* polymorphism was neither related to the age at menarche nor to the timing of reproduction. Our findings are consistent with results from a study performed among the Tsimane community, where there was no association between *ApoE4* and self‐reported age at menarche and age at last reproduction (Trumble et al. 2023). However, Tsimane women with the *ApoE4* allele began reproduction approximately 0.8 years earlier on average (Trumble et al. 2023).

*ApoE4* allele contributes to increased cholesterol levels, which suggests that the *ApoE* gene may influence the metabolism of sex hormones (Finch and Sapolsky 1999). Cholesterol serves as a precursor in the production of steroid hormones such as estrogens and testosterone, supporting the idea that *ApoE* polymorphism may play a role in reproductive processes (Finch and Sapolsky 1999). Indeed, our previous study in a Polish population showed that young women carrying at least one *ApoE4* allele produced 20% more progesterone in their menstrual cycles than women with other genotypes (Jasienska et al. 2015). However, while elevated hormone levels may lead to a greater chance of becoming and staying pregnant (Kumar and Magon 2012), reproduction is influenced not only by sex hormone levels, but also by many other biological, social, and cultural factors (van Exel et al. 2017).

It has been suggested that the *ApoE* impact on fertility may depend on pathogen exposure, and therefore, the association between *ApoE4* and fertility may be influenced also by environmental factors (van Exel et al. 2017). Thus, *ApoE4* may promote fertility in challenging environments with high pathogen exposure (van Exel et al. 2017). *ApoE4* may predispose the organism to fight infections (Oria et al. 2010; Toniutto et al. 2010). This could explain why the *ApoE4* allele still exists in most populations, even though it can have negative health consequences later in life.

Moreover, diet and access to resources may potentially influence the association between *ApoE* and fertility. Women in our population have relatively high energy intake (Nenko and Jasienska 2009) contrary to Tsimane women, who have poorer nutritional status, especially in postreproductive years (Trumble et al. 2023). These differences are evident by comparing the body mass index (BMI). Women participating in our study had an average BMI of 30.1 kg/m^2^, while Tsimane women, whose age range was broader (13–90 years), had an average BMI of only 23.3 kg/m^2^ (Trumble et al. 2023). It is possible that lipid‐regulating factors that can positively influence hormone levels may be more important in resource‐limited situations and might be irrelevant in less limited resource conditions (Lipson and Ellison 1996).

Additionally, nutritional status may affect the timing of menarche and the quality of menstrual cycles (Jasienska 2003). The age at first reproduction is one of the key predictors of reproductive success (Bumpass et al. 1978). A shorter time to first reproduction indicates a higher likelihood of conception, which is strongly linked to good nutritional status and elevated reproductive hormone levels (Jasienska 2003). This effect is particularly pronounced in high‐fertility populations (Jasienska 2003).

There are many factors that may be associated with fertility beyond a single gene polymorphism, including environmental and lifestyle factors, which can also influence gene function and its impact on reproduction (Lozupone et al. 2023). Environmental exposures such as climate, air pollution, and contact with industrial chemicals like pesticides can impair female reproductive functions (Zhirnov et al. 2024). Lifestyle choices, including physical activity, diet, and substance use, may also play a critical role in shaping fertility patterns (Słojewska et al. 2025; Zhirnov et al. 2024). Stress management and maintaining a healthy weight are also crucial, as obesity negatively impacts reproductive health (Kelly‐Weeder and Cox 2007).

One of the strengths of this study is the relatively large sample size of well‐nourished women characterized by high variability in fertility (number of children varied from 0 to 13). Furthermore, the population studied is traditional, with minimal use of contraceptive methods. This allows us to provide a valuable insight into factors related to fertility in a population with limited access to modern methods of contraception. Additionally, our study included only women who completed their reproduction, eliminating biases related to ongoing fertility. The data used in our study are comprehensive, allowing for a detailed analysis of reproductive history, including not only the number of children but also other key reproductive events.

Our findings need to be understood in the context of several limitations. In our study, only 16 (4%) women had two copies of the *ApoE4* allele. However, previous studies have shown that carrying even one *ApoE4* allele could affect women's fertility (Corbo, Scacchi, et al. 2004; Corbo, Ulizzi, et al. 2004; Jasienska et al. 2015; Trumble et al. 2023; van Exel et al. 2017). In our study, 98 (27%) women had at least one *ApoE4* allele. Additionally, as our study included only women who survived to older ages, selective survival cannot be excluded, especially since *ApoE4* carriers present increased cardiometabolic burden and higher mortality (von Berg et al. 2024), this factor should be considered when interpreting our results. Individuals with poorer health may have died earlier and therefore were not captured in the analysis, which could lead to biased results and should be considered when interpreting our findings.

It is also possible that postreproductive women may not accurately recall the timing of various reproductive events (e.g., age at menarche) as the data were collected in a self‐reported manner. However, whenever possible, we verified the obtained information (e.g., age at birth, age at marriage, dates of birth of the children) in parish records or official documents issued by a government. Moreover, study assistants were trained to cross‐check the data during the interviews. Although self‐reported age at menarche is often considered to have limited reliability due to the long recall period, previous research suggested that such data can still be valid. A study on middle‐aged women demonstrated a strong positive correlation (*r* = 0.79, *p* < 0.001) between the recalled age at menarche and the age recorded in original childhood medical records (Must et al. 2002).

## 5 Conclusion

In conclusion, our results indicate that neither *ApoE4* nor *ApoE3* genotype was associated with age at menarche, timing of reproduction, fertility (measured by number of children), and interbirth interval in a population of postreproductive women. The effect of *ApoE* gene on fertility may be partially obscured or outweighed by other, more influential factors such as other genes, environmental conditions, or lifestyle choices (Lozupone et al. 2023).

One key question that remains is why the *ApoE4* allele, often considered deleterious due to its association with negative health outcomes in older age (Farrer et al. 1997), persists in industrialized populations. It is possible that *ApoE4* confers reproductive advantages, but only under specific ecological or environmental conditions such as high pathogen exposure or limited resource availability.

Overall, the growing body of research highlights the need for further studies in diverse populations, particularly those with higher fertility rates, to better understand the mechanisms linking *ApoE*, fertility, and women's reproductive health.

## Author Contributions

P.B., A.G., and G.J. conceptualized the analyses. P.B. performed formal analyses, created visualizations, and drafted the manuscript. I.N., A.G., and M.K. contributed to investigation and methodology. G.J. designed the study and supervised the project. All authors reviewed and approved the final manuscript.

## Funding

This study was supported by the National Science Center (NN404273440 to G.J.); the Ministry of Science and Higher Education (Poland) (IdP2011000161 and N43/DBS/000068 to G.J.); and Salus Publica Foundation.

## Disclosure

This article was written without the use of AI.

## Ethics Statement

The study was performed in accordance with the Declaration of Helsinki. The study received approval from the Jagiellonian University Bioethics Committee (KBET/5/B/2010 and KBET/328/B/2012).

## Consent

All participants provided written, informed consent.

## Conflicts of Interest

The authors declare no conflicts of interest.

## Supporting information


**Data S1:** ajhb70260‐sup‐0001‐Supinfo.pdf.

## Data Availability

The data that support the findings of this study are available on request from the corresponding author. The data are not publicly available due to privacy or ethical restrictions.
