# Supplementary material for: Apolipoprotein E Polymorphism and Reproductive Patterns Among Postreproductive Women
Source: Am J Hum Biol. 2026 Apr 18;38:e70260. doi: 10.1002/ajhb.70260 (PMC13090747; doi:10.1002/ajhb.70260)
Supplement: Supplementary file 1 — Data S1: ajhb70260‐sup‐0001‐Supinfo.pdf. [file AJHB-38-e70260-s001.pdf]

Supporting Information

Apolipoprotein E polymorphism and reproductive patterns among postreproductive women

Paula Bartecka[1,2], Andrzej Galbarczyk[1,3,*], Magdalena Klimek[1], Ilona Nenko[1], Grazyna Jasienska[1]

[1] Department of Environmental Health, Institute of Public Health, Faculty of Health Sciences, Jagiellonian University Medical College, 8 Skawińska Street 31-066 Krakow, Poland

[2] Doctoral School of Medical and Health Sciences, Jagiellonian University Medical College, 16 Łazarza Street 31-530 Krakow, Poland

[3] Lise Meitner Research Group BirthRites - Cultures of Reproduction, Max Planck Institute for Evolutionary Anthropology, Deutscher Pl. 6, 04103 Leipzig, Germany

* Correspondence: Andrzej Galbarczyk (agalbarczyk@gmail.com)

Supporting Information for GLM estimates regarding all covariates

Table S1. Comparison of parameters of reproductive history among groups of women with none and at least one *ApoE4* allele regarding all covariates. Differences among genotypes were tested by General Linear Model.

	Age at menarche (years)			Age at first reproduction (years)			Number of children			Mean interbirth interval (months)			Age at last reproduction (years)		
	**F**	**p**	η_p^2	**F**	**p**	η_p^2	**F**	**p**	η_p^2	**F**	**p**	η_p^2	**F**	**p**	η_p^2
ApoE 4 yes/no	0.052	0.820	< 0.001	0.007	0.932	< 0.001	0.068	0.794	< 0.001	2.495	0.115	0.008	0.445	0.505	0.001
year of birth	0.085	0.771	< 0.001	0.474	0.492	0.001	11.853	0.001	0.034	0.037	0.848	< 0.001	12.414	< 0.001	0.036
education	5.130	0.024	0.015	1.546	0.215	0.005	7.603	0.006	0.022	0.923	0.337	0.003	3.142	0.077	0.009
age at marriage	-	-	-	809.908	< 0.001	0.705	-	-	-	-	-	-	-	-	-
age at first birth	-	-	-	-	-	-	23.358	< 0.001	0.064	5.202	0.023	0.016	35.109	< 0.001	0.095

Note: η_p^2 *—partial eta squared*

Table S2. Comparison of parameters of reproductive history among groups of women with none, one, or two *ApoE4* alleles regarding all covariates. Differences among genotypes were tested by General Linear Model.

	Age at menarche (years)			Age at first reproduction (years)			Number of children			Mean interbirth interval (months)			Age at last reproduction (years)		
	**F**	**p**	η_p^2	**F**	**p**	η_p^2	**F**	**p**	η_p^2	**F**	**p**	η_p^2	**F**	**p**	η_p^2
ApoE 4 genotypes	0.047	0.954	< 0.001	0.275	0.760	0.002	0.037	0.964	< 0.001	1.457	0.234	0.009	0.568	0.567	0.003
year of birth	0.099	0.753	< 0.001	0.360	0.549	0.001	11.733	0.001	0.033	0.072	0.789	< 0.001	12.916	< 0.001	0.037
education	5.096	0.025	0.015	1.569	0.211	0.005	7.585	0.006	0.022	0.910	0.341	0.003	3.170	0.076	0.009
age at marriage	-	-	-	809.286	< 0.001	0.705	-	-	-	-	-	-	-	-	-
age at first birth	-	-	-	-	-	-	23.293	< 0.001	0.064	5.197	0.023	0.016	35.009	< 0.001	0.095

Note: η_p^2 *—partial eta squared*

Table S3. Comparison of parameters of reproductive history among groups of women with none and at least one *ApoE3* allele regarding all covariates. Differences among genotypes were tested by General Linear Model.

	Age at menarche (years)			Age at first reproduction (years)			Number of children			Mean interbirth interval (months)			Age at last reproduction (years)		
	**F**	**p**	η_p^2	**F**	**p**	η_p^2	**F**	**p**	η_p^2	**F**	**p**	η_p^2	**F**	**p**	η_p^2
ApoE 3 yes/no	0.487	0.486	0.001	0.001	0.974	< 0.001	0.457	0.500	0.001	0.295	0.587	0.001	1.006	0.317	0.003
year of birth	0.116	0.734	< 0.001	0.471	0.493	0.001	12.146	0.001	0.034	0.042	0.839	< 0.001	12.838	< 0.001	0.037
education	4.993	0.026	0.015	1.541	0.215	0.005	7.830	0.005	0.023	1.015	0.314	0.003	3.191	0.075	0.009
age at marriage	-	-	-	809.173	< 0.001	0.705	-	-	-	-	-	-	-	-	-
age at first birth	-	-	-	-	-	-	23.519	< 0.001	0.065	5.135	0.024	0.016	34.794	< 0.001	0.094

Note: η_p^2 *—partial eta squared*

Table S4. Comparison of parameters of reproductive history among groups of women with none, one, or two *ApoE3* alleles regarding all covariates. Differences among genotypes were tested by General Linear Model.

	Age at menarche (years)			Age at first reproduction (years)			Number of children			Mean interbirth interval (months)			Age at last reproduction (years)		
	**F**	**p**	η_p^2	**F**	**p**	η_p^2	**F**	**p**	η_p^2	**F**	**p**	η_p^2	**F**	**p**	η_p^2
ApoE 3 genotypes	0.365	0.694	0.002	0.001	0.999	< 0.001	0.311	0.733	0.002	1.514	0.221	0.009	1.294	0.276	0.008
year of birth	0.128	0.720	< 0.001	0.469	0.494	0.001	12.163	0.001	0.035	0.054	0.816	< 0.001	12.908	< 0.001	0.037
education	4.936	0.027	0.015	1.534	0.216	0.005	7.738	0.006	0.022	1.040	0.309	0.003	3.113	0.079	0.009
age at marriage	-	-	-	804.384	< 0.001	0.704	-	-	-	-	-	-	-	-	-
age at first birth	-	-	-	-	-	-	23.593	< 0.001	0.065	5.607	0.018	0.017	34.207	< 0.001	0.093

Note: η_p^2 *—partial eta squared*
